# Successfully controlling intrusive memories is harder when control must be sustained

**DOI:** 10.1080/09658211.2017.1282518

**Published:** 2017-02-21

**Authors:** Kevin van Schie, Michael C. Anderson

**Affiliations:** a Department of Clinical Psychology, Utrecht University, Utrecht, Netherlands; b Institute of Psychology, Erasmus University Rotterdam, Rotterdam, Netherlands; c MRC Cognition and Brain Sciences Unit, University of Cambridge, Cambridge, UK

**Keywords:** Inhibitory control, intrusive memories, involuntary retrieval, retrieval suppression, memory inhibition

## Abstract

After unpleasant events, people often experience intrusive memories that undermine their peace of mind. In response, they often suppress these unwanted memories from awareness. Such efforts may fail, however, when inhibitory control demands are high due to the need to sustain control, or when fatigue compromises inhibitory capacity. Here we examined how sustained inhibitory demand affected intrusive memories in the Think/No-Think paradigm. To isolate intrusions, participants reported, trial-by-trial, whether their preceding attempt to suppress retrieval had triggered retrieval of the memory they intended to suppress. Such counter-intentional retrievals provide a laboratory model of the sort of involuntary retrieval that may underlie intrusive memories. Using this method, we found that longer duration trials increased the probability of an intrusion. Moreover, on later No-Think trials, control over intrusions suddenly declined, with longer trial durations triggering more relapses of items that had been previously been purged. Thus, the challenges of controlling retrieval appear to cause a decline in control over time, due to a change in state, such as fatigue. These findings raise the possibility that characteristics often true of people with psychiatric disorders – such as compromised sleep, and increased demand on control – may contribute to difficulties in suppressing intrusive memories.

Memories of unpleasant life events sometimes intrude into awareness. When this happens, people often seek to limit awareness of the unwelcome reminding by stopping the retrieval process. Evidence indicates that when people consistently suppress retrieval in this manner, suppressed items grow increasingly difficult to recall on later occasions (for reviews, see Anderson & Hanslmayr, 2014; Anderson & Huddleston, 2012). Suppression not only reduces retention on measures of voluntary retrieval, but also on involuntary forms of retrieval such as the tendency for memories to intrude into awareness in response to reminders (Benoit, Hulbert, Huddleston, & Anderson, 2014; Levy & Anderson, 2012), free association (Hertel, Large, Stück, & Levy, 2012), perceptual priming (Gagnepain, Henson, & Anderson, 2014; Kim & Yi, 2013), and implicit association tests (Hu, Bergström, Bodenhausen, & Rosenfeld, 2015). These findings suggest that people can recruit cognitive control mechanisms to adaptively regulate intrusive memories.

Whether this type of memory adaptation is possible, however, may hinge on other conditions that could compromise people’s efforts to implement suppression, such as the total time over which the suppression of an unwanted thought must be sustained. Indeed, most people are familiar with how difficult it can be to sustain attention for long periods of time and to avoid distraction, particularly when the potential for distraction is significant. In the present article, we examined these putative difficulties in the context of memory control. In particular, we examined whether counter-intentional retrievals of the sort that could underlie naturally occurring intrusive memories grow more likely both with sustained challenge to inhibitory control mechanisms, and with fatigue.

## Involuntary memories and their control

The current investigation builds on two distinct, but complementary bodies of research concerning involuntary memory on the one hand, and memory control, on the other. Research on involuntary memory focuses on processes that trigger memories to come to mind automatically and reflexively, and whether they differ from intentional retrieval. Involuntary memories have usually been studied with diaries or questionnaires, in which participants write down how and when autobiographical memories were cued and what their content was (Berntsen, 1996; Bradley, Moulin, & Kvavilashvli, 2013; Kvavilashvili & Mandler, 2004; Mace, 2004). Recently, involuntary memories have been studied in controlled environments (Clark, Mackay, & Holmes, 2013). In one paradigm, participants perform an undemanding vigilance task, while they need to ignore short cue phrases (e.g., “crossing the road”) on a screen (Schlagman & Kvavilashvili, 2008). Whenever an involuntary autobiographical memory came to mind, participants paused the vigilance task and reported their memory and what triggered it. After completing the vigilance task participants reported other characteristics of the involuntary memories, such as emotional valence and whether that memory was general or specific. One week later, participants *voluntarily* recalled autobiographical memories after seeing cue phrases. In two experiments with this paradigm, Schlagman and Kvavilashvili (2008) showed that involuntary memories were retrieved almost twice as quickly as voluntary memories and that these memories were more likely to be specific. This suggests that involuntary memories spring to mind quickly and without effort. However, how people control involuntary memories when they arise has remained largely unstudied in this research (e.g., for exceptions, see Brewin & Smart, 2005; Geraerts, Merckelbach, Jelicic, & Smeets, 2006).

Complementing this work is a largely separate literature examining how people voluntarily control memory. Research on memory control tries to isolate mechanisms by which people might prevent or interrupt the reflexive retrieval of unwelcome memories when confronted with reminders, as well as the consequences of engaging those mechanisms for the retention of suppressed traces. This form of memory control is often studied with the Think/No-Think (TNT) paradigm (Anderson & Green, 2001), which examines people’s ability to suppress the episodic retrieval process to control intrusive memories. In the TNT paradigm, people are repeatedly prompted with cues to previously studied associates and are asked to either retrieve the memory (hereinafter, Think trials), or to instead stop its retrieval and exclude the unwanted memory from awareness (hereinafter, No-Think trials). Participants study a third set of items that are neither recalled nor suppressed (hereinafter, baseline items). Afterwards, memory for all pairs is tested by an unexpected memory test to determine how retrieval suppression influenced later retention of the excluded memories.

A large body of research shows that suppressing retrieval impairs later recall of No-Think items compared to baseline items, an effect known as suppression-induced forgetting (Anderson et al., 2004; Anderson & Green, 2001; Anderson & Hanslmayr, 2014; Anderson & Huddleston, 2012). Suppression-induced forgetting arises with many types of stimuli, including word pairs, face–scene pairs (Depue, Curran, & Banich, 2007), face–word pairs (Hanslmayr, Leipold, Pastötter, & Bäuml, 2009), word– object pairs (Gagnepain et al., 2014; Kim & Yi, 2013), and pairs comprising words and nonsense shapes (Hart & Schooler, 2012). Suppression-induced forgetting has even been observed with autobiographical experiences especially for event details (Hu et al., 2015; Noreen & MacLeod, 2013; 2014; Stephens, Braid, & Hertel, 2013). Importantly, suppression-induced forgetting occurs for emotional materials, including negatively valenced words and scenes (e.g., Catarino, Küpper, Werner-Seidler, Dalgleish, & Anderson, 2015; Chen et al., 2012; Depue et al., 2007; Hertel & McDaniel, 2010; Joormann, Hertel, Brozovich, & Gotlib, 2005; Kim, Oh, Kim, Sim, & Lee, 2013; Küpper, Benoit, Dalgleish, & Anderson, 2014; Lambert, Good, & Kirk, 2010; LeMoult, Hertel, & Joormann, 2010; Marzi, Regina, & Righi, 2014; Murray, Anderson, & Kensinger, 2015; Murray, Muscatell, & Kensinger, 2011; van Schie, Geraerts, & Anderson, 2013). Evidence suggests that suppression-induced forgetting can be produced in two ways – either as an aftereffect of processes that directly suppress retrieval, or by processes that promote self-distraction, such as thought substitution (e.g., Benoit & Anderson, 2012; Bergström, de Fockert, & Richardson-Klavehn, 2009b; Hertel & Calcaterra, 2005; Hertel & McDaniel, 2010; Küpper et al., 2014). Overall, this body of work on suppression-induced forgetting indicates that suppressing unwanted thoughts about past events can be successful, in contrast to findings observed with Wegner’s thought suppression procedure (Wenzlaff & Wegner, 2000), with the latter procedure suggesting that thought suppression can be counterproductive (a point to which we return in the Discussion).

Research on retrieval suppression assumes that presenting a cue triggers an automatic retrieval process that elicits associated memories, unless effort is made to proactively prevent or reactively interrupt this process. In essence, this work assumes the form of automatic retrieval process studied in research on involuntary memories, without which there would be no need for control. Moreover, because research on retrieval suppression focuses on how people suppress *unwanted* involuntary retrievals, its mission is to address intrusive memories per se, a subclass of involuntary memories that are both unwanted and perseverative (Kvavilashvili, 2014). Although the assumption of involuntary retrieval in the TNT task is plausible, evidence for it has, until recently, been indirect. Next we discuss direct evidence for intrusive retrievals, and the control processes needed to counter them.

## Measuring intrusions and their demands on inhibitory control

To examine the assumption that involuntary retrievals arise during retrieval suppression, Levy and Anderson introduced a trial-by-trial judgment task during the TNT phase of the TNT paradigm, asking participants’ to report their phenomenal experience of whether the unwanted memory intruded into awareness (Benoit et al., 2014; Hellerstedt, Johansson, & Anderson, 2016; Levy & Anderson, 2012). This method was modelled after the trial-by-trial introspection method developed in research on selective attention (Corallo, Sackur, Dehaene, & Sigman, 2008; Sergent & Dehaene, 2004; Sergent, Baillet, & Dehaene, 2005). After every Think and No-Think trial, participants judged the extent to which the target came to mind by pressing one of three buttons associated with responses “never”, “briefly”, and “often”. Using this procedure, Levy and Anderson (2012) found that involuntary retrievals of the unwanted memory occurred often; up to 60% of the time on the first suppression attempt. With repeated suppressions, however, participants reduced these retrievals substantially (on average, down to 30%). Levy and Anderson also found that a steeper reduction in involuntary retrievals over repeated suppressions (i.e., a steeper negative slope) predicted greater suppression-induced forgetting on the final test, suggesting that controlling involuntary retrieval engages processes that trigger forgetting (see also Hellerstedt et al., 2016).

The foregoing findings suggest that online trial-by-trial reports may provide a useful new method to isolate automatic retrieval processes of key interest in research on involuntary retrieval (Berntsen, 2010). Indeed, the tendency for memories to enter awareness in direct opposition of participants’ intensive efforts to prevent a retrieval from occurring provides a conceptually precise operational definition of involuntary retrieval, given that such retrievals are counter-intentional, a hallmark feature of automaticity. Unlike most diary studies of involuntary retrieval, which only establish a lack of clear intention to retrieve a memory (warranting the phrase “non-intentional”), counter-intentional retrievals justify the use of the phrase “involuntary”. Counter-intentional retrievals may provide an informative laboratory model of the retrieval processes that underlie intrusive memories, which are involuntary retrievals that are both unwanted and potentially disruptive (Kvavilashvili, 2014). In line with their counter-intentional nature, we therefore refer to the involuntary retrievals reported by our participants as “intrusions” (Levy & Anderson, 2012) that may be relevant to clinical reports of intrusive memories (a point to which we will return in the Discussion).

Several studies have used this intrusion reporting procedure to study the cognitive control demands present during intrusive memories, and the brain systems that are engaged. In general, retrieval suppression increases activation in the (right) dorsolateral prefrontal cortex (hereinafter, DLPFC; Anderson et al., 2004; Benoit & Anderson, 2012; Depue et al., 2007; Depue, Burgess, Willcutt, Ruzic, & Banich, 2010; Gagnepain et al., 2014; Levy & Anderson, 2012), an area linked to inhibitory control over motor responses (see Levy & Wagner, 2011 for a meta-analysis). Attempting to stop retrieval in this manner reduces hippocampal activity, a finding often taken to reflect inhibitory processes that interrupt retrieval activity that might otherwise allow an intrusion to occur. Critically, Levy and Anderson (2012) found that during No-Think trials on which an intrusion was experienced, hippocampal down-regulation was especially pronounced, and this retrieval-related down-regulation predicted later forgetting far better (*r* = .7) than did hippocampal activity during No-Think trials when participants did not experience an intrusion (*r* = −.07). Benoit et al. (2014) further found that although suppression generally engaged right DLPFC, trials with intrusions triggered elevated activity compared to trials without intrusions, consistent with the possibility that cognitive control is up-regulated. Recently, Hellerstedt et al. (2016) found that during retrieval suppression, No-Think trials that are accompanied by intrusions show evidence that the unwanted item is briefly retrieved into working memory and then is rapidly excluded (i.e., ERP indices of working memory appear and then are quickly truncated); in contrast No-Think trials that are not accompanied by intrusions do not show ERP evidence of the unwanted item entering working memory. Together, these findings demonstrate that intrusion reports can isolate involuntary retrieval in the TNT procedure, and that these intrusions can be linked to objective neural indices of elevated cognitive control.

If suppressing intrusions requires elevated cognitive control, this process should be resource demanding. This raises the possibility that diminished cognitive control resources may trigger difficulty dealing with intrusions. Consistent with this possibility, research on individual differences in inhibitory control ability supports the idea that compromised cognitive control leads to difficulties in suppressing intrusive memories. For example, individual differences in stop-signal reaction time (i.e., a measure of how quickly a person can inhibit a motor action, indexing inhibitory control) predict the ability to successfully forget emotionally negative pictures (Depue et al., 2010). Moreover, participants with major depressive disorder show reduced suppression-induced forgetting compared to control participants (Joormann, Hertel, Lemoult, & Gotlib, 2009), as do participants with anxiety disorder, post-traumatic stress disorder, and attention deficit disorder (ADD) (Catarino et al., 2015; Depue et al., 2010; Marzi et al., 2014). Given this evidence, task or state-dependent strains on cognitive control might also be associated with increased incidence of intrusive memories. We discuss these possibilities next.

### Task and state-dependent challenges to controlling memory intrusions

Task conditions and transitory states can place a burden on cognitive control and may influence how effectively retrieval suppression can be engaged to control intrusive memories. For instance, the effort required to control episodic retrieval may be especially pronounced when attention must be sustained on a reminder for a longer time. Sustained attention to a reminder provides more time for the cue to drive retrieval of associated memories, increasing the risk of involuntary retrieval. Moreover, sustained attention to a reminder would require continuous vigilance and engagement of inhibitory control mechanisms to counter the tendency for cues to elicit a memory. Thus, the need to sustain attention to reminders is a task state variable that arguably places a heavier burden on inhibitory control. Consistent with this possibility, Lee, Lee, and Tsai (2007) observed significantly less suppression-induced forgetting on long duration No-Think trials (5 seconds) than on short duration trials (3 seconds). Lee et al. (2007) argued that sustaining inhibition over longer durations increases the chances of cognitive control failure and, consequently, intrusions. With more intrusions, they argued, later recall could be enhanced, owing to reinforced encoding of intruding traces. However, Lee et al. had no way of measuring the frequency of intrusions to verify their assumption of increased inhibitory demand. In the present study, we therefore used Lee et al.’s manipulation of trial duration, which we adjusted to include short (2.5 seconds) and long (5 seconds) durations for both Think and No-Think trials, and combined this with the intrusion report method. Based on Lee et al.’s claim, we hypothesised that longer trial durations would trigger greater demands on inhibitory control, yielding a higher probability of intrusions on long trials compared to short trials. This greater intrusion frequency may arise from increased input to retrieval stemming from sustained viewing of the cue, or instead from lapses in the task set governing inhibitory control, due to fatigue, either of which would indicate greater demands on control.

To test this hypothesis, we used the intrusion report method during the TNT phase to determine whether participants report significantly more intrusions during longer trials than during shorter ones. If sustaining control over time is demanding, we should also find indications of decreased efficacy of control over a longer time scale spanning the entire 35-minute duration of the TNT phase. This putative waning efficacy may be detectable in the patterns of intrusions over the eight suppression attempts on an item. Prior work has established a monotonic decline in reported intrusions over repeated suppression attempts during the TNT phase, possibly reflecting the tendency for prior inhibitions to accumulate in their impact on suppressed traces (Benoit et al., 2014; Hellerstedt et al., 2016; Levy & Anderson, 2012). This accumulation of inhibition may mask, however, a waning efficacy of the control process owing to fatigue. To test this possibility, we examined fine-grained item-by-item, transition patterns in memory control success that arise across consecutive suppression attempts on the same item. For example, by looking at the intrusion histories of individual items we can examine how well a participant’s efforts to suppress retrieval of *a particular item* on one trial then carry forward to the next trial with that same item. This enables us to look at the conditions that lead to successful suppression (i.e., when an intrusion on trial *N* is followed by successful control on trial *N* + 1 with that item, hereinafter referred to as *new successes*), and also conditions that increase the chances of memory control *relapses* (i.e., when successful control on trial *N* is followed later by failed control on trial *N* + 1 with that particular item). If sustaining control over a much longer time scale leads to fatigue, two patterns should emerge. First, although new successes should increase over early repetitions, they should, as fatigue sets in, ultimately decline, indicating a reduced ability to down-regulate intrusions from one repetition to the next. Second, an initial period in which participants can reduce control relapses over repetitions should be followed by an increase in relapses towards the end of the TNT phase, as participants grow fatigued. This pattern would reflect diminishing ability to suppress intrusions in a way that has a lasting impact on later trials. Finally, we would expect relapses to be generally more likely during longer duration trials, owing to the greater demand those trials place on inhibitory control.

## Method

### Participants

Participants were recruited from Erasmus University Rotterdam undergraduate psychology classes via the Erasmus Psychology Study Pool. To be eligible for the study, participants had to be between 18 and 35 years of age and fluent in Dutch. Additionally, they reported never to have been diagnosed with ADD or Attention Deficit Hyperactivity Disorder (ADHD) or to be colour blind. Forty-four undergraduates participated for course credit. Data from three participants were excluded because of scoring errors made by the experimenter during the experimental procedure; one participant was excluded because of failing to reach learning criterion, leaving a final sample of 40 (*M* = 20.28 years, *SD* = 1.87; 25 females and 15 males).

### Materials and design

The stimuli consisted of 60 randomly combined neutral Dutch cue-target word pairs (e.g., BARREL – NUN), which were equally divided over five word groups. The cues and targets were drawn from three previous studies (Anderson et al., 2004; Anderson & Green, 2001; van Schie et al., 2013), and were also, in part, newly constructed. For newly constructed pairs, we selected emotionally neutral words from a large dataset of Dutch words by Moors et al. (2013). We used relatively short noun words with a maximum length of 10 letters and 3 syllables. Selected words typically are concrete noun words (e.g., Stomach, Desk). For as far as possible we tried to select words that did not have a strong association with other words in the stimulus set. Such an association was limited to the targets and independent probes only. For each pair’s target we selected an associated word from the association database from the University of Leuven (www.kuleuven.be/semlab) to serve as an independent probe (see Table 1 in the Appendix for all pairs’ cue, target, and independent probes). These independent probes provide secondary cues for the target words (e.g., Cloister N____ for Nun). These cues enable us, on the final test, to test all targets twice, once with the studied cue, once with an independent cue. Though not crucial to the purpose of the current study, testing targets in two ways provided converging evidence for the generality of suppression-induced forgetting across cues, supporting the involvement of inhibition in controlling retrieval (Anderson & Green, 2001). Importantly, there was no evidence for the five experimental word groups to differ statistically on ratings (taken from Moors et al., 2013) of valence, arousal, power, and age of acquisition for cue, target, and independent probe; nor did they differ on word length, word frequency, and association strength between target and independent probe. Eighteen additional cue-target pairs served as fillers. Filler pairs were used to train participants on the TNT task (see description of the TNT phase in The TNT Procedure below).

Word groups were rotated through conditions (Baseline, Think short, Think long, No-Think short, and No-Think long), so that each word group was presented equally often in each condition.

### TNT procedure

The experiment was run with E-prime 2.0 (Psychology Software Tools, Pittsburgh, PA). During the procedure, the experimenter was present to provide instructions for each phase and verbal encouragement when necessary. Vocal responses were scored out of the participant’s sight.

#### General instructions

At the outset of the experiment, participants were told that they were about to participate in an experiment on attention, and that their ability to ignore distraction would be assessed. They were told that they would learn pairs of words to be used in the attention test, and that they would need to ignore associations in memory. No reference was made to a final memory test for the words at any point in the procedure, to ensure that participants took the retrieval suppression task seriously and did not covertly rehearse suppression words during the TNT phase.

#### Learning phase

The total set of 60 critical pairs was learned in three subsets of 20. Each subset of 20 critical items had an equal representation of items from each counterbalancing set. In the first set, 26 pairs (20 critical plus 6 filler) were presented one by one in white font on a black background in the middle of a screen for 4000 ms with a 400 ms intertrial interval (ITI). Pseudo-randomised test-feedback cycles followed in which participants were instructed to verbally recall each target when presented with its cue. Cues disappeared after 3500 ms or upon verbal response. The correct target was displayed in blue for 1000 ms followed by a 400 ms ITI regardless of the participant’s answer. If at least 50% of the experimental target words were recalled correctly, participants progressed immediately to the second subset of words; if not, the test was repeated once. Regardless of the percentage correct on set repetition, the participant continued to the second subset of 26 words, for which the same procedure ensued. After this, they continued to the third and final subset. The phase concluded with an integrated test (without any feedback) for pairs from all three sets. Participants repeated this phase if they did not reach the 50% cut-off. If participants did not reach the cut-off on their second test, they were excluded from further participation.

#### TNT phase

On each trial, a fixation cross appeared for 400 ms followed by a cue presented for either 2500 ms for short trials or 5000 ms for long trials. For Think cues, presented in green, participants were asked to retrieve its target silently; for No-Think trials, presented in red, they received direct suppression instructions, asking them to suppress retrieval of the target *without* substituting something else for the target. Cue words from *Baseline items*, though learned in the study phase, did not appear in this phase, and thus provided a baseline estimate of memory for pairs, given that neither suppression nor retrieval took place during the TNT phase. After each cue word, a rating scale appeared on the screen and participants had 1500 ms to report the extent to which the target entered awareness during that trial; the scale included values of “never”, “briefly”, and “often”, and participants indicated their response by pressing a corresponding button with their dominant hand. Trials were separated by a 400 ms ITI.

The TNT phase consisted of four blocks separated by 45-second breaks. Each block contained 96 cues, representing 12 cues in each Instruction × Duration combination, each repeated twice. No more than three cues with the same instruction or duration were displayed consecutively. Across all blocks, each cue was repeated eight times. Before the critical TNT phase, participants performed two practice blocks with 24 filler trials: 12 No-Think and 12 Think trials. The first practice block was conducted without the rating scale for either Think or No-think items; the second practice block added the ratings for all trials.

#### Final test phase

After the TNT phase, participants’ memory was assessed unexpectedly using two types of tests, the order of which was counterbalanced across participants. In the same probe test, participants were presented with the pair’s cue (e.g., BARREL – ____) and in the independent probe test, with a word associated to the target along with a letter stem (CLOISTER – N____). All items were assessed in the same probe and in the independent probe test. Participants were given up to 10 seconds to recall the target. Trials were separated by a 400 ms ITI. A short practice test on fillers preceded these tests.

#### Post-experimental questionnaire

After the test, participants filled out a post-experimental questionnaire rating their use of different strategies during No-Think trials to verify that participants followed our direct suppression instructions. All strategies were rated on a 5-point scale ranging from 0 *never* to 4 *always* indicating use of each strategy and whether they intentionally did not follow experimental instructions, and how awake or sleepy they felt before they started the experiment. We further measured *intentional* non-compliance with No-Think instructions to ensure that all participants had genuinely tried to exclude memories from awareness (Hertel & Calcaterra, 2005). Intentional non-compliance was measured with three questions: (a) When I saw the red cue word, I quickly checked to see if I remembered the target word, (b) After a red cue word went off the screen, I checked to see if I still remembered the target word, and (c) When I saw a red cue word, I thought about the target word that went with it in an effort to improve my memory for that word pair. These questions were rated on a 5-point scale ranging from 0 *never* to 4 *very frequently*.

## Results

All analyses are based only on pairs for which participants recalled the target on the final learning test (Anderson et al., 2004). Overall, learning performance on this test was sufficiently high: 72.44% (*SE* = 1.66). To retain power in all subsequent analyses, slight violations of sphericity were corrected with either Greenhouse–Geisser (.70 ≥ *ε* < .75) or Huynh–Feldt corrections (ε ≥ .75). In case of severe violations (.70 < *ε*) a multivariate test statistic (Pillai-Bartlett trace; *V*) is reported. In all analyses counter-balancing condition was included as a between-subjects factor to account for item effects, and nonsignificant results of this factor (or its interactions) are not reported.

### Final recall performance

Though intrusion reports from the TNT phase were our primary interest, we replicated prior findings concerning suppression-induced forgetting on the final test. Replicating past work, suppression affected final recall, as evidenced by poorer recall of target words in the suppress conditions overall (No-Think short *M* = .83, *SE* = .013; No-Think long *M* = .841, *SE* = .013) compared to recall of target words from Baseline pairs (*M* = .861, *SE* = .011), *F*(1, 35) = 4.337, *p* = .045, $\eta_{\text{p}}^{2}$ = .11. There was no reliable difference in recall between short duration and long duration suppression conditions, *F*(1, 35) = .378, *p* = .543, nor were there interactions with test type (i.e., same probe or independent probe test), *F*s < 1.3. None of the foregoing suppression-induced forgetting effects (or comparisons of short vs long No-Think) interacted with test-order counter-balancing (all *p*s > .25).

### Replicating key patterns in control over intrusions during the TNT task

Our main hypothesis was that longer trial durations would be accompanied by a greater number of intrusions than would shorter trial durations, as measured by our trial-by-trial intrusion reports collected during the TNT phase. However, we first assessed whether we replicated past work on reports of conscious awareness in the TNT phase (e.g., Levy & Anderson, 2012), by conducting a 2 (condition: Think/No-Think) × 8 (repetition) ANOVA, with short and long duration trials averaged per condition. If participants reported that a memory came to mind “briefly”, or “often” during a No-Think trial, we considered this an intrusion; if they reported that a memory “never” came to mind during the trial, we considered this a non-intrusion. Participants also made the same judgments after Think trials, in which case the awareness of the memory was the desired goal, so this would not be considered an “intrusion” per se. But the reports in the two conditions can be compared nonetheless, to quantify variation in mnemonic awareness as a function of task goals.

**Figure 1:**
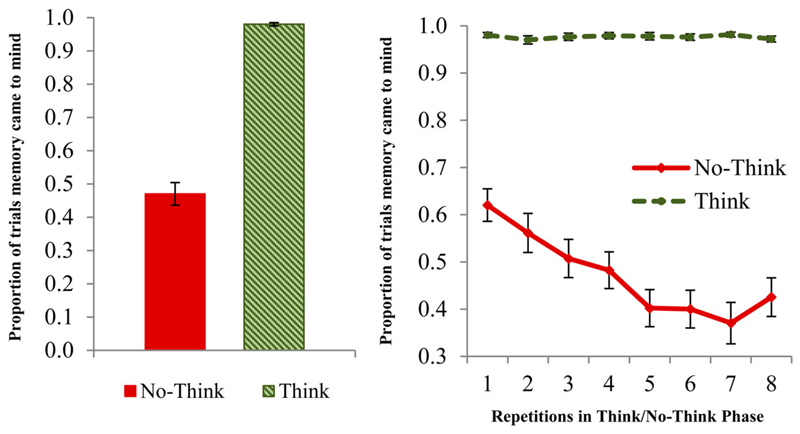
Proportion of trials during which the memory came to mind displayed for No-Think trials and Think trials (left panel), and the proportion of intrusions over repetitions in the TNT Phase (right panel) for No-Think and Think trials. Error bars represent standard errors of the mean.

Directly comparing reports of mnemonic awareness across the Think and No-Think conditions, we observed an exceptionally robust main effect, *F*(1, 35) = 212.839, *p* < .001, $\eta_{2}^{\text{p}}$ = .859, demonstrating that people experienced target memories entering awareness far less often during No-Think trials (*M* = .47, *SE* = .034) than during Think trials (*M* = .98, *SE* = .005). Thus, according to participants’ phenomenal reports, they showed a remarkable overall ability to voluntarily control episodic retrieval. Nevertheless, intrusions still were experienced on a significant proportion (approximately half of the time) of No-Think trials (see Figure 1, left panel).

Reports of intrusions declined substantially across suppression repetitions. This pattern is reflected in a robust Repetition × Condition interaction, *V* = .602, *F*(7, 29) = 6.255, *p* < .001, $\eta_{\text{p}}^{2}$ = .602. This interaction was followed up by one ANOVA for Think trials and one for No-Think trials. These showed that reports of awareness did not vary over repetitions for Think trials *V* = .142, *F*(7, 29) = .689, *p* = .681, but were reduced substantially from the first No-Think repetition (*M* = .62, *SE* = .034) to the eighth one (*M* = .43, *SE* = .041), *V* = .599, *F*(7, 29) = 6.196, *p* < .001, $\eta_{\text{p}}^{2}$ = .599. Thus, participants grew increasingly effective at suppressing intrusions of No-Think items as repetitions progressed, and remained highly effective at bringing Think trials to mind across all repetitions (see Figure 1, right panel). These results strongly confirm findings from prior neuroimaging studies using this trial-by-trial intrusion scale (Benoit et al., 2014; Hellerstedt et al., 2016; Levy & Anderson, 2012). Increasing success at controlling intrusions may reflect some combination of the accumulation of inhibition on suppressed traces over repetitions, improved skill at stopping retrieval in general, and a passive decline in the accessibility of No-Think items over time, though prior work clearly indicates a significant contribution of inhibitory control (e.g., Benoit et al., 2014; Levy & Anderson, 2012).

### Sustaining inhibitory control continuously is demanding

To test whether confronting a reminder during No-Think trials for longer periods of time is more demanding than confronting a reminder for shorter periods of time, we included trial duration (short vs. long) as a within-subjects factor in our analysis of intrusion reports, and conducted a 2 (duration: Short/Long) × 8 (repetition) ANOVA. This analysis revealed a highly reliable difference in the frequency of intrusions between short (*M* = .44, *SE* = .035) and long duration (*M* = .50, *SE* = .035) No-Think trials, *F*(1, 35) = 22.856, *p* < .001, $\eta_{\text{p}}^{2}$ = .395. Thus, longer trial durations likely imposed a greater burden on inhibitory control, as reflected in the increased probability of intrusions, an effect that did not interact with repetition, *F* < 1. This trial duration effect also was apparent when intrusion reports for No-Think trials were broken down into “brief” and “often” responses. Brief intrusions arose more often during long (*M* = .46, *SE* = .03) than during short trials (*M* = .42, *SE* = .03), *t*(39) = 3.015, *p* = .005. Similarly, participants reported that an intrusion “often” came to mind with greater frequency during long (*M* = .04, *SE* = .009) than during short trials (*M* = .02, *SE* = .004), *t*(39) = 2.339, *p* = .025. Consistent with our hypothesis concerning trial durations, “never” responses were mirrored to “briefly” and “often” responses; there were more “never” responses on short trials (*M* = .56, *SE* = .03) compared to long trials (*M* = .50, *SE* = .03), *t*(39) = 4.149, *p* < .001.

**Figure 2:**
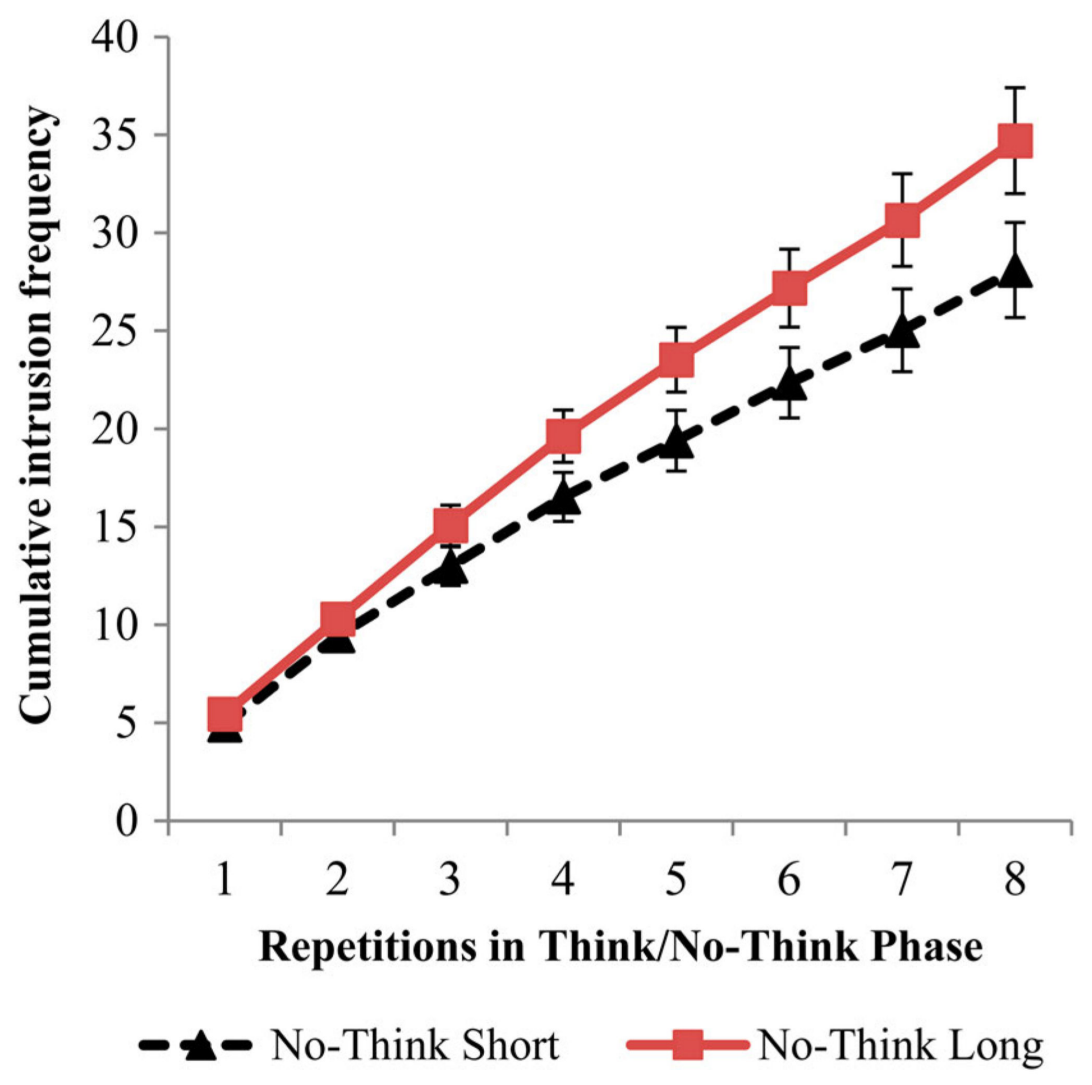
Cumulative intrusion frequency per repetition for short and long duration No-Think trials, which reflects the sum of reported intrusions on each repetition and all preceding repetitions. Error bars represent standard errors of the mean.

Further evidence for greater demand on control processes can be plainly seen in the cumulative intrusion functions for our duration conditions. In these functions, the number of intrusions on each repetition is the sum of intrusions reported during that repetition and all preceding repetitions. A 2 (duration: Short/Long) × 8 (repetition) ANOVA showed that on long duration trials there were more intrusions overall, *F*(1, 35) = 14.393, *p* = .001, $\eta_{\text{p}}^{2}$ = .291, and a significant Duration × Repetition interaction showed that the number of intrusions accumulated more quickly over No-Think repetitions for long duration compared to short duration trials, *V* = .529, *F*(7, 29) = 4.658, *p* = .001, $\eta_{\text{p}}^{2}$ = .529 (see Figure 2). Moreover, although people often were able to successfully control intrusions (i.e., make intrusions of an item stop entirely for all of its remaining No-Think trials), it took significantly more trials to achieve this goal for long duration No-Think trials. To quantify this, we computed, for each item, the average number of No-Think repetitions until the last intrusion was experienced. For longer duration trials, the average serial position of the final intrusion occurred later during the TNT phase (*M* = 5.95, *SE* = .24), than it did for short duration trials (*M* = 5.32, *SE* = .28), *t*(39) = 3.754, *p* = .001. Thus, increasing trial duration made it harder to succeed quickly at suppression, likely reflecting the increased demand placed on inhibitory control mechanisms.

**Figure 3:**
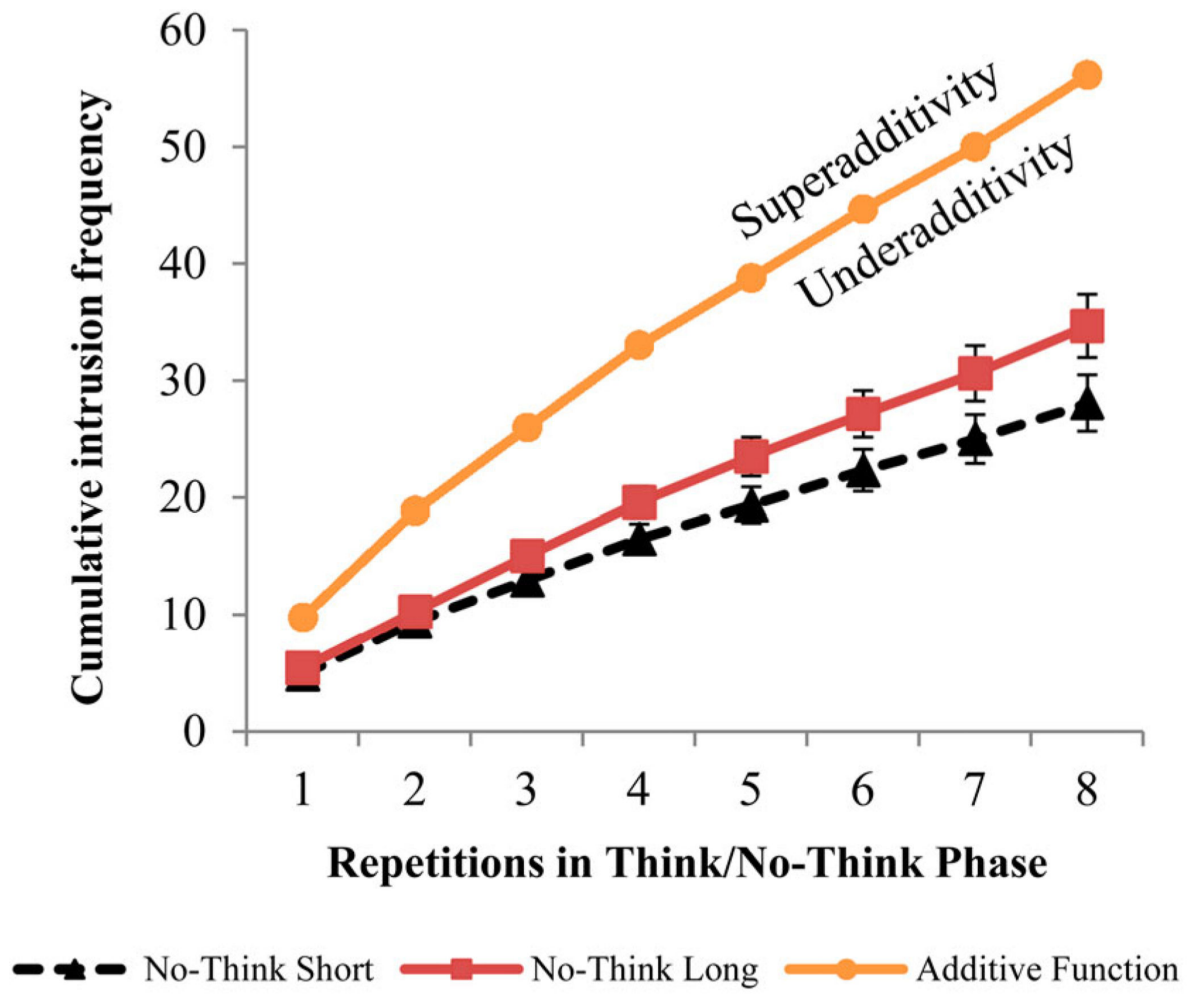
Additive function for cumulative intrusion frequency, which is plotted based on the average number of cumulative intrusions in short duration No-Think trials times two. It represents a uniform increase of intrusions with time. The position of the long duration No-Think cumulative intrusions relative to the additive function, shows that the relationship between time and the probability of an intrusion occurring is underadditive (i.e., the No-Think duration line lies below the additive function). Error bars represent standard errors of the mean.

Next, we investigated the nature of the relationship between time and the probability of an intrusion occurring. It is conceivable that if one intrusion is experienced within a short duration No-Think trial (i.e., 2.5 seconds), then two intrusions should be experienced within a long duration No-Think trial (i.e., 5 seconds). This would be classified as an additive relationship, wherein intrusions increase uniformly with the number of seconds (see Figure 3, line with circular markers). Alternatively, the relationship may be superadditive; in this case, the number of intrusions experienced over time is higher than what would be expected based on an additive relationship. Long duration No-Think trials would show more than twice the number of intrusions compared to short duration trials, perhaps reflecting increasing within-trial fatigue. A final possibility is an underadditive relationship, wherein with increasing trial duration, intrusions may still increase, but less than would be expected based on a simple additive relationship. Figure 3 illustrates an exploratory analysis we conducted illustrating that an underadditive relationship best characterises our data. The proportion of intrusions for long duration trials is much less than twice the amount observed during short trials, and the cumulative recall function increases for long trials, but not at a rate that is double the slope of the short trials.

**Figure 4:**
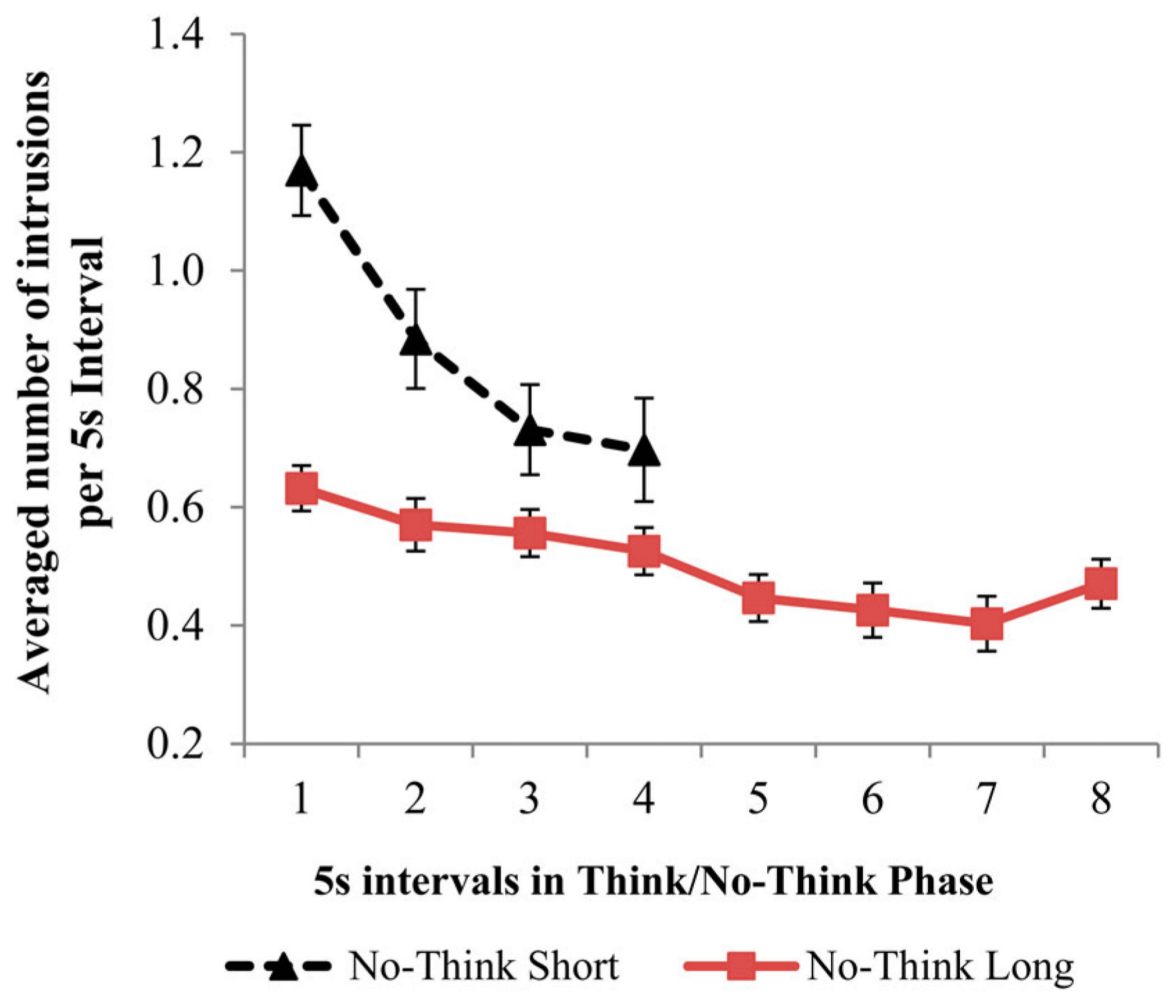
Average number of intrusions per 5-second interval for the short and long duration No-Think conditions. Each 5-second interval for the short duration No-Think condition is the sum of two repetition intervals that were 2.5 seconds originally. Error bars represent standard errors of the mean.

To further characterise the relationship between time and an intrusion occurring, we looked at the number of intrusions per second for short and long duration trials. We estimated the number of intrusions per second by totalling the number of intrusions reported for each item across its eight repetitions, and then dividing by the total number of seconds the participant spent suppressing that item (e.g., 8 suppression attempts × 2.5 seconds per attempt = 20 seconds; or × 5 seconds per attempt = 40 seconds). Remarkably, the number of intrusions per second was much lower in the long (*M* = .10, *SE* = .007) than in the short trials (*M* = .17, *SE* = .01), *t*(39) = 9.067, *p* < .001. To verify this difference was not the result of an overall difference in the total time suppressing unwanted memories in the short (20 seconds of total suppression time) and long trials (40 seconds of total suppression time), we restricted our comparison to the first 20 seconds within the short and long conditions. Thus, for the short condition, the first 20 seconds would cover all 8 repetitions (2.5 seconds × 8 repetitions), whereas for the long condition, it would cover the first 4 repetitions (5 seconds × 4 repetitions). In addition, to illustrate how the number of intrusions *per unit time* changed with repetitions, we compared the development of intrusion rates across the first 20 seconds in the long duration condition and the first 20 seconds in the short duration condition. To do this, we divided the first 20 seconds into 4 equal size bins of 5 seconds each. For the long duration condition, these bins were simply the first four 5 suppression trials, each of which was 5 seconds long. For the short duration condition, these 5-second bins were each composed by aggregating over two consecutive 2.5-second long trials on a given item. For each of these 5-second bins, we then summed the intrusions observed within it. This enables us to plot intrusion rate (intrusions per 5-second bin) across four time bins matched for duration in the short and long conditions. A 2 (duration: Short/Long) × 4 (repetition interval) ANOVA showed that even with this more precise matching of total time, the average number of reported intrusions per 5 second bin in long condition (*M* = .57, *SE* = .07 or.11 intrusions per second) remained lower than in the short condition (*M* = .87, *SE* = .034 or.17 intrusions per second), *F*(1, 35) = 44.561, *p* < .001, $\eta_{\text{p}}^{2}$ = .56. Interestingly, a significant Duration × Repetition interaction showed that though intrusions per each 5 second intervals in the short condition decreased with repetition, they remained higher at the fourth repetition, compared to long condition intervals, *F*(3, 105) = 10.46, *p* < .001, $\eta_{\text{p}}^{2}$ = .23 (see Figure 4).

Finally, we considered the possibility that the foregoing conclusions about underadditivity and intrusion rate might have arisen because we failed to adequately consider trials that contained multiple intrusions, which occurred slightly more often on long duration than short duration trials (4% of the trials instead of 2%). Specifically, the foregoing analyses do not distinguish between trials where participants report a single brief intrusion (the subject responded “briefly” on the intrusion scale) and trials where participants report having experienced more than one intrusion (the subject responded “frequently”), because, in either case, a participant was simply classified as having an intrusion on that trial. It is possible that if we gave credit for trials with more than one intrusion, the conclusions might differ. To address this, we recomputed all analyses using a graded scale (0 for non-intrusions, 1, for a brief intrusion, 2 for frequent intrusions). All of the foregoing analyses turned out the same, with differences between conditions in fact growing slightly larger and more reliable, not less (intrusions per second in the long versus short conditions, *M* = .11 and.18 respectively, *p* < .001; in the analysis using only the first 20 seconds,124 versus 184, respectively (.62 vs. 92 intrusions per 5-second bin), *p* < .001, $\eta_{\text{p}}^{2}$ = .58). The similarity in the analyses reflects the fact that multi-intrusion trials were rare (only 3% of reports) and thus could not exert much influence on the data. Thus, our conclusions about underadditivity and intrusion rates seem unlikely to derive from this analysis choice. Nevertheless, although “frequent” responses were a very small part of the data, it would be desirable, in future studies, to quantify the number of intrusions on such trials more precisely to yield a more accurate measurement.

### Repeated inhibitory control is demanding

If applying inhibitory control is effortful, the quality of that effort may decline as blocks progress, particularly in the later blocks and under more strenuous conditions. To test this we calculated a transition score between every two sequential repetitions of each item (e.g., the second suppression attempt of item X, and the third attempt on X, irrespective of other items that may have intervened between those repetitions of X), and determined in what proportion of transitions an intrusion was followed by a non-intrusion (i.e., new successes), and a non-intrusion was followed an intrusion (i.e., relapses). Successes give us a measure of how well one’s efforts to suppress retrieval of a particular item carry forward to later trials with that item; this may indicate persisting aftereffects of suppression on the item in question. Relapses, on the other hand, suggest that one’s prior successes at controlling awareness did not produce lasting effects on the suppressed item, and possibly signal a lapse in applying control mechanisms to that item.^1^

**Figure 5:**
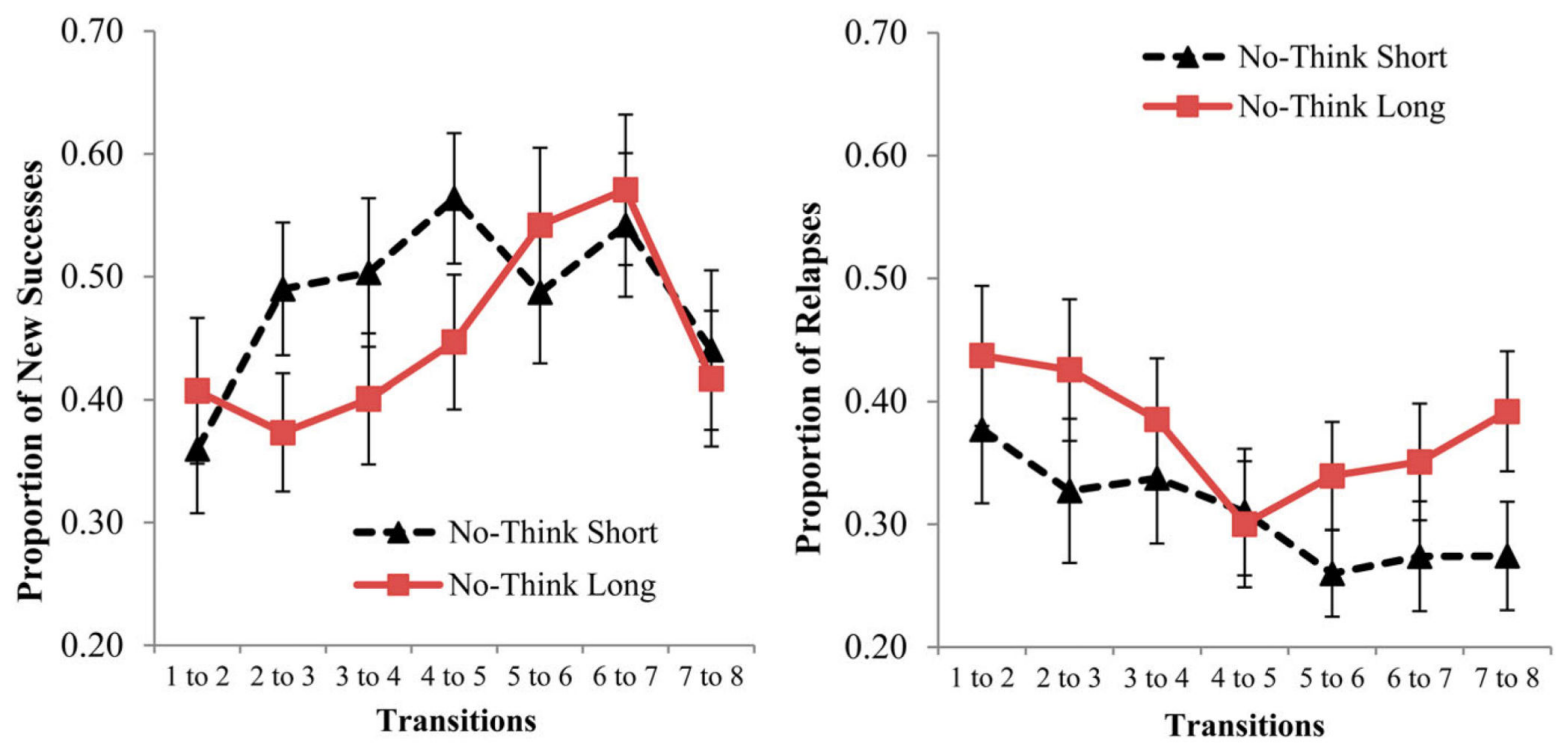
Proportion of new successes (left panel) and relapses (right panel) per repetition-to-repetition transition for short and long duration No-Think trials. New successes reflect the transition from an intrusion to non-intrusion, while relapses represent the transition from a non-intrusion to an intrusion. Pooled means and standard errors of the mean are presented.

For successes, there was an effect for Repetition which shows that the probability of a new success (i.e., transitions from intrusions to non-intrusions) increased reliably over repetitions at first, but then the pattern reverses in later blocks displaying a clear quadratic relationship, *χ*^2^(35) = 89.191, *p* < .001 (see Figure 5, left panel). There was no reliable difference in the probability of new successes between short- and long duration conditions, *χ*^2^(35) = −4.719, *p* > .05, nor was there an interaction of No-Think trial duration with repetition, *χ*^2^(210) = −6.787, *p* > .05. Taken together, these findings indicate that the probability of achieving a new successful control declined suddenly towards the end of the TNT task, consistent with the possibility that participants were growing fatigued during the final repetitions. Alternatively, the drop in new successes may reflect greater difficulty in suppressing the remaining intrusions, which may be the most persistent and demanding items.

In contrast to new Successes, Relapses did show sensitivity to trial duration; relapses were significantly more frequent in the long duration condition (*M* = .376, *SE* = .029) compared to the short duration condition (*M* = .309, *SE* = .031), *χ*^2^(35) = 63.55, *p* = .002 (see Figure 5, right panel). There was no main effect of repetition, *χ*^2^(108) = −4.137, *p* > .05 or an interaction of duration with repetition, *χ*^2^(210) = −19.126, *p* > .05. This suggests that when trial duration was longer, it was more difficult for suppression to induce a persisting impact that carried forward to the next suppression trial for a given item.

We also sought to relate the foregoing patterns to participants’ subjective reports of alertness. Post-experimentally, we asked participants to rate, on a 5-point scale, their alertness at the outset of the experiment. Ratings on this scale were uniformly high (*M* = 4.18), so there unfortunately was little variability in initial alertness that could be related to subsequent intrusions. Thus, participants having lower alertness at the outset of the study did not reliably predict increased relapse probability (when examined overall, *r* = −.16; when focusing on the second half of TNT phase, *r* = −.26), nor did it predict decreased new successes (overall, *r* = .01, second half, *r* = −.05). However, the three participants who reported unusually compromised alertness (scores of 1 or 2) did have a far higher relapse probability (*M* = .63) than the remaining subjects with relatively normal alertness (*M* = .37), especially when considering the second half of the TNT phase (Low = .68, Remainder = .31).

### Compliance

All (100%) of participants reported that they *often* or *always* used strategies consistent with direct suppression instructions, such as staring intently at the cue word, repeating the cue word silently, or letting their mind go blank in response to the cue word. Strategies consistent with thought substitution, such as generating a word, thought or sound in response to the No-Think cue, were never (0%) reported as being used *often* or *always*. A minority of participants (45%) reported they *rarely* or *sometimes* used a thought substitution strategy. Thus, participants largely complied with our instructions to directly suppress retrieval of No-Think targets. Moreover, non-compliance with No-Think instructions was low; the majority of participants indicated *never* or *rarely* trying to intentionally violate No-Think instructions, respectively 87.5%, 95%, and 97.5% for our three compliance questions (see Methods).

## Discussion

The findings of the current study support the conclusion that control over involuntary retrieval is more challenging if it must be sustained for longer periods of time. To characterise how challenging it was for participants to control retrieval, we used a trial-by-trial intrusion report method developed in prior work to document participants’ phenomenal experience of intrusions during retrieval suppression. These reports have been used successfully to identify distinctive hemodynamic and electrophysiological signatures of inhibitory control over memory (Benoit et al., 2014; Hellerstedt et al., 2016; Levy & Anderson, 2012). Using intrusion reports, we tested two main hypotheses. First, if needing to continuously suppress episodic retrieval in response to a prepotent reminder for longer intervals taxes inhibitory control processes to a greater extent, more intrusions should be observed during long duration trials (5 seconds) than short duration trials (2.5 seconds). Increased intrusion frequency during longer trials may be produced either by more sustained input to the retrieval process owing to attention to the cue, or to lapses in task set maintenance with longer intervals, either of which would make stopping retrieval more challenging. The results provide clear support for this duration effect: participants reported experiencing intrusions with a greater probability during longer suppression trials than during shorter ones. This pattern arises primarily from an increase in the number of trials on which a single intrusion occurred, rather than an increase in the number of intrusions per trial, though a relatively modest increase in multi-intrusion trials also was evident in the data. The increase in the probability of an intrusion occurring was present across all suppression repetitions. These findings demonstrate that being exposed to reminders for longer durations increases the challenge posed to controlling retrieval. This increased challenge is also reflected in the fact that, on average, it took participants 5.95 trials to fully control all intrusions of an item for long trials, but only 5.32 repetitions for short duration trials.

Beyond the effects of trial duration, we also hypothesised that there would be a gradual deterioration of the ability to control intrusions when control must be maintained over very long intervals. In a typical TNT task, participants engage in multiple blocks of memory control that, taken together, may last between 30 and 60 minutes, and we have previously noted that participants can become fatigued after such intervals (Anderson & Huddleston, 2012). If sustaining control in this manner is challenging, evidence for increasing burden may be evident in intrusion reports, which may reveal participants’ declining ability to control retrieval towards the end of our TNT task. We predicted that although this pattern would be seen in changes in overall intrusion frequency, it would be particularly striking in our sequential dependency analyses of the intrusion data. The data partially support these predictions. On the one hand, we found the predicted deterioration in new successes (cases in which an intrusion on trial *N* is followed by a non-intrusion on trial *N*+1) towards the end of the TNT phase, (see Figure 5); on the other hand, the predicted quadratic trend in relapses (cases in which a non-intrusion on trial *N* is followed by an intrusion on trial *N*+1), while numerically present for long duration trials (see Figure 5), was not reliable. Long duration trials were, nevertheless associated with more relapses than were short duration trials, consistent with the notion that they impose greater demands on inhibitory control.

One interpretation of these cross-block changes in the success of inhibitory control is that participants suffered from increasing fatigue after 35 minutes of performing a demanding retrieval suppression task. This possibility is broadly consistent with the sensitivity of executive functioning to sleep deprivation, which shows that sleep deprivation increases the difficulty in inhibiting inappropriate responses (Chuah, Venkatraman, Dinges, & Chee, 2006; Drummond, Paulus, & Tapert, 2006). For example, a recent study using a Go-No Go task showed that mental fatigue indeed gives rise to delayed motor inhibition (Kato, Endo, & Kizuka, 2009). Because there is overlap between the systems involved in motor and memory inhibition (Anderson & Weaver, 2009; Depue, Orr, Smolker, Naaz, & Banich, 2015), mental fatigue may affect memory inhibition in a similar fashion. However, although fatigue provides a plausible explanation for these cross-block patterns, the present data cannot distinguish fatigue from other factors that could also have changed over blocks, any of which may account for poor task performance, including changes in mood, and, perceived novelty of the task (i.e., boredom). There is evidence indicating, for example, that the sort of cognitive conflict that triggers cognitive control is an aversive state (Inzlicht, Bartholow, & Hirsh, 2015) that people seek to avoid or minimise such cognitive effort (Kool, McGuire, Rosen, & Botvinick, 2010). Thus, it is possible that engaging in inhibitory control over a sustained interval may induce negative mood. Future studies should seek to distinguish these accounts, perhaps through a detailed characterisation of fatigue, affect, and interest throughout the task. Our current measurement of alertness was restricted to alertness at the outset of the task, and (fortunately or unfortunately) revealed nearly all participants to feel fully alert.

Despite the clear increase in reported probability of intrusions for longer duration trials, a separate question remains whether control is more *efficiently* exercised if it is done over shorter or longer intervals. One way to quantify the efficiency of control is to compare the rate of intrusions per second across the two conditions. Interestingly, by this measure, longer duration trials were more efficient, in that they are associated with significantly fewer intrusions per second (.10) than were shorter duration trials (.17). This conclusion held when we focused on the first 20 seconds of suppression for each condition (note that there the short duration condition involved a total of only 20 seconds – i.e., 2.5 seconds across 8 trials) and compared “time bins” of the same size (i.e., 5 second bins – which is a single trial in the long duration condition, and two trials in the short duration condition). Thus, the true probability of intrusions as a function of time suppressing was much lower for long duration trials (.11 intrusions per second, or .57 per 5 seconds) than it was for shorter duration trials (.17 intrusions per second, or .87 intrusions per 5 seconds), suggesting that participants got better results from their efforts. Indeed, as can be seen in Figure 4, the increased intrusion rate for shorter duration trials is particularly pronounced during early bins, and shrinks rapidly with repeated effort. In contrast, for longer duration trials, the intrusion rate is uniformly lower, and appears relatively constant over time bins, with little evidence for improvement in rate over repetitions. This increased efficiency with longer trial durations is likely to explain the pattern of underadditivity we observed in our data: we found that 5-second trials were characterised by far fewer intrusions than would be expected if these trials were equivalent to two 2.5-second trials, and inhibition efficacy was strictly a function of the time devoted to the task.

Why would control over intrusions be more efficient if it is applied for longer intervals? One likely explanation is that when performing two 2.5-second trials versus one 5-second trial, participants need to reinstate the control operation twice in the former instance. Transitioning into a No-Think trial surely involves switching of task sets (Allport, Styles, & Hsieh, 1994), and, until the suppression task set is successfully implemented by the participant on a given trial, they run the risk of being reminded of the associated memory. Put differently, the race between two parallel processes – the involuntary retrieval process on the one hand, and the controlled implementation of the inhibition task set on the other – may be lost by the inhibition process some fraction of the time, yielding an intrusion. Thus, when comparing the same total amount of time (5 seconds), there are two occasions on which the task set must be implemented in the short duration condition and only one in the longer duration condition. Long duration trials enable participants to simply sustain an already implemented control operation and devote more time to application of control and less to its initial implementation. If this interpretation is correct, then the steeper decline in the rate of intrusions per second during short duration trials over consecutive time bins suggests (relative to that observed during long duration trials; see Figure 4), that short duration trials, while less efficient, are more effective in driving some form of durable improvement. This could, in principle reflect more effective buildup of inhibition on suppressed traces during short duration trials.

The current study demonstrates the importance of trial duration in experiments on motivated forgetting. In previous experiments there has been considerable between-experiment variation in No-Think trial duration; anywhere between 2 seconds (Bergström, de Fockert, & Richardson-Klavehn, 2009a) and 6 seconds (Marx, Marshall, & Castro, 2008), or even 8 seconds (Wang, Cao, Zhu, Cai, & Wu, 2015). This may have inadvertently affected the number of experienced intrusions and, with it, the size of suppression-induced forgetting. Indeed our effects of trial duration are consistent with the interpretations offered by Lee et al. (2007), who speculated that long trial durations during the TNT task are associated with a greater burden on inhibitory control. In support of this idea, Lee et al. (2007) observed less suppression-induced forgetting on long duration (5 seconds) trials compared to short duration (3 seconds) trials. Because the current study added a trial-by-trial intrusion report measurement, we were able to show that long duration trials were indeed more likely to elicit the need for control; these trials were accompanied by more intrusions, a faster accumulation of intrusions over repetitions, and a larger number of trials until control over intrusions fully succeeded. Unlike Lee et al. (2007), however, we did not find a clear distinction between short and long duration No-Think trials in suppression-induced forgetting, as assessed with the final recall test. This finding suggests that participants’ reports of intrusions provide a more sensitive measure for assessing the effects of the suppression process, compared to a voluntary final recall test. It is possible, for example, that control processes engaged during explicit recall on the final test may undo the effects of suppression (especially on partially suppressed items) in a way that does not arise when accessibility of No-Think items is assessed by the propensity for an item to involuntarily intrude.

Our evidence that “relapses” in controlling intrusive memories are more likely with longer trial durations may shed light on another frequently used paradigm investigating the nature and controllability of intrusive thoughts. Studies using the “white bear” paradigm have found that suppressing thoughts about a white bear continuously for 5 minutes often paradoxically increases the presence of the suppressed thoughts as measured by a similar self-report process to the one used here (Wegner, Schneider, Carter, & White, 1987). Work with this paradigm has suggested that suppression is not only an ineffective method of mental control, but may also be a counterproductive one (Wenzlaff & Wegner, 2000). Although there are a variety of important ways in which the TNT and White Bear paradigms differ (see Anderson & Huddleston, 2012 for a discussion with hypotheses), the current experiment suggests that a key difference may lie in the total amount of time participants must continuously suppress. Our data suggest that purging unwanted thoughts of white bears may be more successful when purging attempts are implemented in short spikes of activity. “White bear” experiments may yield a counterproductive pattern partially as a result of a heavy burden placed on inhibitory control arising from the need to suppress an unwanted thought for a very long period of time. The greater effectiveness of suppression attempts on shorter distributed trials may be related to the benefits of spaced practice over massed practice (Donovan & Radosevich, 1999).

To the extent that our findings provide evidence that sustained control (whether on a trial or session level) can be fatiguing, they may have implications for psychopathology. It has been put forward that inhibitory deficits may be the reason that intrusive memories or thoughts can be observed in a wide-range of psychopathologies, such as PTSD (flashbacks), obsessive–compulsive disorder (obsessions), or depression (rumination) (e.g., Anderson & Hanslmayr, 2014; Catarino et al., 2015; Fawcett et al., 2015; Hertel & Gerstle, 2003; Joormann et al., 2009; Marzi et al., 2014). Given the prevalence of sleep disorders in psychiatric conditions (Wulff, Gatti, Wettstein, & Foster, 2010), fatigue is especially important to identify, because fatigued patients may be less likely to engage inhibitory control effectively. As a result they will experience more intrusive memories than those who are well rested, perhaps further deteriorating their psychiatric condition.

The intrusion report method used here identifies and quantifies the frequency of counter-intentional retrievals that are triggered by strong reminders to an unwanted memory. Because these retrievals occur despite participants’ efforts to stop them, and because they recur whenever cues are presented, we have argued that the processes triggered may be related to those underlying intrusive memories in psychopathology. Consistent with this view, recent discussions of how intrusive memories differ from involuntary retrievals more broadly have emphasised qualities such as their perseverative nature and how they arise, despite being unwanted (Kvavilashvili, 2014). However, clinically relevant intrusive memories also are emotionally intense and stressful, and can be highly disruptive to the individual experiencing them, unlike the intrusions studied here. These differences are clearly important and may signify fundamentally different retrieval mechanisms from the ones studied here. Our view, however, is that the emotional nature of naturally occurring intrusive memories and their personal significance likely function to substantially increase the frequency with which people “self-cue” the internal triggers that lead to the intrusions, via their emotional state or thoughts, but that, once cued, the involuntary retrieval processes are fundamentally similar to the ones at work here. However, regardless of one’s theoretical assumptions, the current paradigm provides a useful tool for comparing the intrusive qualities of a wide variety of memories – whether neutrally, negatively, or positively valenced, or whether autobiographical or laboratory-based – and testing the extent to which the cognitive and neural mechanisms triggered are similar or distinct.

In conclusion, the current findings provide evidence supporting the view that intrusive memories can be controlled, but that it is harder to do so when control must be sustained. These findings suggest that the need to sustain attention to reminders indeed places a heavier burden on inhibitory control. Our novel approach of analysing sequential dependencies between intrusion repetitions confirmed this and additionally showed that needing to sustain control over very long intervals decreases successful suppression of unwanted memories. The finding that sustained engagement compromises inhibitory control is also especially relevant for understanding intrusive thoughts and memories in psychopathologies, particularly to the extent that such deficits arise from fatigue. Indeed, fatigue could, in principle, be a key factor contributing to failed memory control in psychiatric conditions. In future research, phenomenal reports of intrusions of the sort used here may provide a helpful method for studying, in-depth, how intrusive memories are controlled in different psychiatric conditions. Indeed, the particular sequential dependency patterns operationalised here (e.g., relapse probability) may provide new measures that could predict vulnerability to intrusive symptomatology, above and beyond individual variation in suppression-induced forgetting (Levy & Anderson, 2008).

## Supplementary Material

Table A1 Cue, target, and independent probe for the words in the five experimental groups and words in the filler group.

## Note

1 We used multiple imputation in SPSS (Version 22) for transition scores in successes and relapses, because 5.63% of the data were missing. Little’s MCAR test shows no identifiable pattern exists for the missing data, *χ*^2^(583) = 596.58, *p* = .339. Next, 25 datasets were imputed (automatic imputation method). Regular ANOVAs for main or interaction effects were performed on the imputed datasets and all 25 *F*-values were pooled using the R package miceadds, which produces a *χ*^2^ statistic per effect to test for significance (Robitzsch, Grund, Henke, & Robitzsch, 2016).
